# Parallel Finite Element Model for Multispecies Transport in Nonsaturated Concrete Structures

**DOI:** 10.3390/ma12172764

**Published:** 2019-08-28

**Authors:** Okpin Na, Yunping Xi

**Affiliations:** 1 R&D Division, Hyundai E&C, Gyeonggi-do 14102, Korea; 2 Civil, Environmental, and Architectural Engineering, University of Colorado at Boulder, Boulder, CO 80309, USA

**Keywords:** multispecies, diffusion, Nernst–Planck equation, parallel finite element model, coupled hygro-chemo, concrete degradation

## Abstract

The chloride-induced corrosion of steel reinforcement embedded in concrete is undoubtedly one of the most important durability problems of reinforced concrete structures. The chloride ions as well as other ionic species (Na^+^, Ca^2+^, K^+^, OH^−^) come from various deicing salts and they are transported from the environment into concrete. To investigate the transport mechanism of the multispecies, complex scientific methods and accurate mathematical models are needed. The purpose of this study is to develop a more robust mathematical model and better computational technique to characterize the coupled effect of ionic transport mechanisms as well as the influence of interaction of ionic species. The new mathematical model was developed based on the Nernst–Planck equation and null current condition to solve the ionic-induced electrostatic potential, and the model was implemented by a parallel finite element algorithm. The verification of mathematical model was done by comparing the model prediction with experimental results for ionic transport in saturated concrete. The comparisons showed good results. The model prediction of the multispecies transport in partially saturated concrete demonstrated that the ionic species dissolved in pore solution could be carried by the moisture movement and pressure gradient. Therefore, the multispecies transport model based on the parallel finite element method is effective, accurate, and can be used for solving the partial differential equations for ionic species transport in concrete.

## 1. Introduction

Concrete is the most widely used construction material in the world because of its availability and good performance. Relatively low tensile strength and low ductility of concrete lead to the necessity of steel reinforcement in concrete structures. Reinforced concrete structures have a long-term durability problem that is the corrosion of steel reinforcement in concrete. This problem is especially severe under the service conditions such as roadways and parking structures where deicing chemicals are used in the winter and marine structures where the structures are in direct contact with seawater. In these reinforced concrete structures, the corrosion of steel has been considered to be caused mainly by the chloride ions in the deicers or seawater, called chloride-induced steel corrosion. However, the chloride ion is not the only ion penetrating into the concrete, several other ionic species in the environment penetrating into the concrete together with the chloride. For examples, sodium chloride (NaCl), calcium chloride (CaCl_2_), and magnesium chloride (MgCl_2_) are commonly used deicers on highway pavements and reinforced concrete bridges for snow and ice control in winter season, so, Na^+^, Ca^2+^, and Mg^+^ from the deicers can penetrate into concrete structures with the Cl^−^. Frequently, different types of deicers can be used in the same area at the same time and the interactions among the ions have significant effects on the corrosion behavior of the steel in concrete and thus on the service life of reinforced concrete structures [1,2,3]. 

For several decades, many researches have been performed on the modeling of chloride penetration into concrete [1,2,3,4,5,6,7,8,9,10,11,12,13,14,15,16,17]. The focus of this study is to characterize the transport behavior of chloride and other multispecies in concrete under nonsaturated condition. Advanced mathematical models and parallel computing techniques will be applied. 

Fick’s laws of diffusion are widely used as a basic mathematical equation for the transport of species in a porous medium under the action of a concentration gradient. However, Fick’s laws are insufficient for revealing the mechanism of actual ionic transport by coupling concentration gradient and electroneutrality condition to model the multispecies transport in electrolyte solutions. Thus, for the transport processes including concentration gradients, electrical field, pressure flow, and chemical activity, the flux of ionic species can be expressed by Nernst–Plank equation [5,6,7].

For the simulation of electrochemical multispecies transport in saturated concrete, the mathematical model and finite element (FE) model have been developed with the Nernst–Planck equation related to the ionic interaction of multi-ions in concrete pore solution. This model can be incorporated with influences of boundary conditions, current density, and transport parameters of concrete. In electrical field, the concentration profile of ionic species charged by various current densities can be demonstrated by considering the activity of ions in the solution [8,9]. For the electrochemical chloride removal of concrete, Nernst–plank equation was proposed with the consideration of the effect of electrostatic coupling of charged ions, adsorption, porosity, and tortuosity of pore structure [8,9,10,11,12,13,14]. 

To evaluate the electrical coupling between ions in porous media and to consider the chemical activity effects in non-ideal solution, Nernst–Planck equation can be solved together with Poisson’s equation. Lately, the transport model of ions in nonsaturated cement-based materials was researched in relation with external sulfate or calcium and hydroxide leaching. A mathematical model was developed based on a set of Nernst–Planck and Poisson equation by taking into account the diffusion, electrical coupling, chemical activity, and advection mechanisms in the isothermal condition [15,16,17]. 

Recently, a multispecies transport in a multiphase medium has been developed by considering the effects of aggregates, interface transition zones (ITZs), and ionic binding on chloride penetration in concrete to provide a more accurate prediction of the complex interaction between ions [18,19,20].

As mentioned above, the transport equation of multispecies has been developed with mathematical theories for obtaining analytic solutions. It turns out that finding explicit solution is very difficult due to the complexity of equations and limits of analytical tools. Thus, computational techniques such as finite element method (FEM), finite difference method (FDM), and so on need to be employed [4,21,22,23].

The phenomenon of multi-ions transport occurs usually in a thin layer of concrete structures covering steel bars (called concrete cover, with a thickness of 50–75 mm). In order to capture the distribution profiles of multi-ions in non-steady state in the concrete cover, very small finite elements must be adopted. For predicting the service life of large-scale concrete structures with different environmental conditions, enormous number of elements are needed and each element consists of several nodes and each node includes lots of information [21]. To make the computational model more efficient, parallel computing is one of effective techniques to save computational time [22,23]. As increasingly available parallel computing facilities, the implementation of parallel algorithm for multispecies transportation equations has become easier than before. In this study, a parallel finite element model was implemented by a higher level library, PETSc (Portable, Extensible Toolkit for Scientific Computation) and MPI (Massage Passing Interface) based on C++ program language.

In this study, a mathematical model for the transport of multispecies deicing salts in concrete is presented. The model involves not only the effect of transport mechanism but also the influence of interaction of the ionic species. Furthermore, the coupled effect of moisture and multi-ions diffusion is incorporated in the model. The numerical results are obtained by the parallel finite element method and are verified by available experimental results in saturated concrete. Finally, multispecies transport is predicted in partially saturated concrete.

## 2. Basic Transport Formulation of Unsaturated Concrete

### 2.1. Governing Equation

Concrete can be considered as a composite material with three phases including aggregate, cement paste/mortar, and porosity. The transport of ionic species is negligible in the aggregate because the transport parameters of aggregate are much lower than those for cement paste and mortar. However, a pore system provides the diffusion path to migrate ionic species. Therefore, the transport of multispecies (Cl^−^, Na^+^, Ca^2+^, K^+^, OH^−^) in concrete mainly occurs in the cement paste or mortar and in the pore solutions. Total chloride is the sum of bound and free chloride. The free chlorides are water-soluble and they can attack the passive film on the surface of reinforced rebar. In traditional methods to predict the service life of concrete structures, only chloride ions are taken into account. Even though this way is simple and easy to implement in the computation, it fails to characterize realistically the concrete deterioration mechanisms due to ionic and mineral interactions. To better describe the ionic transport process in a porous media, the governing equation of multispecies transport in partially saturated concrete affected by moisture gradient can be written as follows.

For the flux of each species in the pore solution of saturated concrete, the Nernst–Plank equation is

(1) $$J_{i}=-\left[D_{i}\nabla C_{f-i}+Z_{i}D_{i}\left(\frac{F}{RT}\nabla \phi \right)C_{f-i}\right]$$

where *J_i_* is the flux of ions, *D_i_* is the diffusion coefficient, *C_f–i_* is the concentration of multispecies (Na^+^, Ca^2+^, K^+^, OH^−^, Cl^−^), *Z_i_* is the charge number, *F* is the Faraday’s constant, *R* is the gas constant, *T* is the temperature, *Ф* is the electrostatic potential, and index *i* represents for *i*-th species.

Under an internally induced electric field (no external current is applied), all the ionic species in the pore solution have effect on the electroneutrality condition. In order to solve the system of equations, a supplemental relation is needed to consider the electrical potential caused by the movement of all species. There are three methods to determine the magnitude of *Ф*.

Electroneutrality

(2) $$\sum Z_{i}C=0$$

Null current

(3) $$\sum Z_{i}J_{i}=0$$

Poisson equation

(4) $$\sum Z_{i}C_{i}=-\varepsilon \nabla \cdot \nabla \phi$$

where *ε* is the dielectric constant. These three equations are taken over all ionic species in the solution. Nguyen et al. found that an electrostatic potential could be evaluated in terms of electroneutrality which can simplify the numerical simulation shown in Equation (2) [12]. The electrostatic potential equation based on null current in Equation (3) was mentioned by Li and Page, Truc et al., and Wang et al. [9,10,14]. Poisson’s equation shown in Equation (4) was used by Samson and Marchand to calculate the electrostatic potential [16,17]. In this study, the null current flux assumption was employed to solve Equation (1). For the electro potential Φ, Equation (3) is substituted into Equation (1).

(5) $$\nabla \phi =\frac{RT}{F}\frac{\sum_{i=1}^{N}Z_{i}D_{i}\nabla C_{i}}{\sum_{i=1}^{N}Z_{i}^{2}D_{i}C_{i}}$$

Finally, the flux of multispecies in nonsaturated concrete is,

(6) $$J_{i}=-\left[D_{i}\nabla C_{f-i}+Z_{i}D_{i}\left(\frac{F}{RT}\nabla \phi \right)C_{f-i}+D_{i-H}\nabla H\right]$$

The mass balance equations for each ionic species can be expressed as

(7) $$\frac{\partial C_{t-i}}{\partial t}=-\nabla J_{i}$$

By substituting Equation (1) into Equation (4), yields

(8) $$\frac{\partial C_{t-i}}{\partial t}=-\nabla \left[D_{i}\nabla C_{f-i}+Z_{i}D_{i}\left(\frac{F}{RT}\nabla \phi \right)C_{f-i}+D_{i-H}\nabla H\right]$$

where, $\frac{\partial C_{t-i}}{\partial t}=\frac{\partial C_{t-i}}{\partial C_{f-i}}\cdot \frac{\partial C_{f-i}}{\partial t}+\frac{\partial C_{t-i}}{\partial H}\cdot \frac{\partial H}{\partial t}$.

The moisture transport can be described,

(9) $$J_{H}=-\left[D_{H-i}\nabla C_{f-i}+D_{H}\nabla H\right]$$

where *J_H_* is the flux of relative humidity, *D_H_* is the diffusion coefficient, and *H* is relative humidity.

In the same way, the mass balance equations for moisture is

(10) $$\frac{\partial w}{\partial t}=-\nabla J_{H}=-\nabla \left[D_{H-i}\nabla C_{f-i}+D_{H}\nabla H\right]$$

where, $\frac{\partial w}{\partial t}=\frac{\partial w}{\partial C_{f-i}}\cdot \frac{\partial C_{f-i}}{\partial t}+\frac{\partial w}{\partial H}\cdot \frac{\partial H}{\partial t}$.

Finally, Equations (8) and (10) can be combined and simplified into matrix form,

(11) $$\left[\begin{array}{cc} \frac{\partial w}{\partial H} & \frac{\partial w}{\partial C_{f-i}}\\ \frac{\partial C_{t-i}}{\partial H} & \frac{\partial C_{t-i}}{\partial C_{f-i}} \end{array}\right]\cdot \left\{\begin{array}{c} \frac{\partial H}{\partial t}\\ \frac{\partial C_{f-i}}{\partial t} \end{array}\right\}=\nabla \left[\begin{array}{cc} D_{H-H} & D_{H-i}\\ D_{i-H} & D_{i-i} \end{array}\right]\cdot \left\{\begin{array}{c} \nabla H\\ \nabla C_{f-i} \end{array}\right\}$$

where, $C_{f-i}$ is the concentration of multispecies (Na^+^, Ca^2+^, K^+^, OH^−^, Cl^−^). Total degree of freedom in this matrix form is 6 unknowns.

### 2.2. Material Parameters

In order to numerically solve the multispecies transport problem, material parameters shall be needed as shown in Equations (12) and (13); the moisture capacity (∂w/∂H), ionic binding capacity ($\partial C_{t-i}/\partial C_{f-i}$), humidity diffusion coefficient ($D_{H}$), and chloride diffusion coefficient ($D_{Cl}$) [24].

### 2.3. Capacity and Diffusivity Coefficients of Multispecies in Nonsaturated Concrete

The moisture capacity (∂w/∂H ) of concrete can be determined by the average of the moisture capacities of cement paste and aggregate as proposed by Xi et al. [25,26,27,28,29]. The chloride binding capacity $\left(\partial C_{t-\mathrm{cl}}/\partial C_{f-\mathrm{cl}}\;\right)$ is defined as the ratio between the change of free chloride content and total chloride content. Recently, it was developed by Xi and Bazant [30]. The other species (Na^+^, Ca^2+^, K^+^, OH^−^) capacity is employed as 1 in this study.

(12) $$\left[\begin{array}{cc} \frac{\partial w}{\partial H} & \frac{\partial w}{\partial C_{f-i}}\\ \frac{\partial C_{t-i}}{\partial H} & \frac{\partial C_{t-i}}{\partial C_{f-i}} \end{array}\right]=\left[\begin{array}{cccccc} \frac{\partial w}{\partial H} & 0 & 0 & 0 & 0 & 0\\ 0 & \frac{\partial C_{t-cl}}{\partial C_{f-cl}} & 0 & 0 & 0 & 0\\ 0 & 0 & 1 & 0 & 0 & 0\\ 0 & 0 & 0 & 1 & 0 & 0\\ 0 & 0 & 0 & 0 & 1 & 0\\ 0 & 0 & 0 & 0 & 0 & 1 \end{array}\right]$$

The moisture diffusion coefficient $\left(D_{H-H}\right)$ of concrete based on composite theory derived by Christensen can be calculated by the relationship with the diffusion coefficient of aggregate and the diffusion coefficient of cement paste [31]. The diffusion coefficient of chloride ion $\left(D_{cl-cl}\right)$ in concrete can be estimated using the multifactor method proposed by Xi and Bazant [30]. It depends on the influence of water–cement ratio, composite action of the aggregates, relative humidity, temperature, and free chloride concentration. A detailed derivation of mass balance equation for multispecies including electrical coupling term could be referred to Appendix A.

(13) $$\begin{array}{l} \left[\begin{array}{cc} D_{H-H} & D_{H-i}\\ D_{i-H} & D_{i-i} \end{array}\right]=\\ \left[\begin{array}{cccccc} D_{H-H} & D_{H-cl} & D_{H-Na} & D_{H-K} & D_{H-OH} & D_{H-Ca}\\ D_{cl-H} & D_{cl-cl} & D_{cl-Na} & D_{cl-K} & D_{cl-OH} & D_{cl-Ca}\\ D_{Na-H} & D_{Na-cl} & D_{Na-Na} & D_{Na-K} & D_{Na-OH} & D_{Na-Ca}\\ D_{K-H} & D_{K-cl} & D_{K-Na} & D_{K-K} & D_{K-OH} & D_{K-Ca}\\ D_{OH-H} & D_{OH-cl} & D_{OH-Na} & D_{OH-K} & D_{OH-OH} & D_{OH-Ca}\\ D_{Ca-H} & D_{Ca-cl} & D_{Ca-Na} & D_{Ca-K} & D_{Ca-OH} & D_{Ca-Ca} \end{array}\right] \end{array}$$

### 2.4. Coupling Parameters

The influence of chloride and moisture was described by Ababneh and Xi and Abarr [32,33]. Ababneh and Xi conducted an experimental study on the effect of chloride penetration on moisture transport in concrete [32]. Based on the result, the effect of chloride penetration on moisture transport is significant. Thus, the coupling parameter, $D_{H-cl}$, is not a constant value, but it depends on chloride concentration. Abarr investigated the effect of moisture transport on chloride penetration in saturated concrete [33]. The results indicate that $D_{cl-H}$ increases with increasing of chloride concentration and $D_{cl-H}$ decreases up to zero when the free chloride concentration is small. Therefore, it can be concluded that $D_{cl-H}$ is chloride concentration dependent. In this study, as the coupling parameters, ε and δ for $D_{cl-H}$ and $D_{H-cl}$, 0.19 and 0.52 were used.

(14) $$D_{cl-H}=\varepsilon \cdot cl_{f}$$

(15) $$D_{H-cl}=\delta \cdot cl_{f}$$

However, there have been no experimental data or material models of coupling parameters for other species, Na, Ca, OH, and K. As the first approximation, the coupling parameters of these ionic species can be evaluated proportionally based on the previous research [32,33]. As shown in Equation (16), the parameter considering the effect of moisture on ionic diffusion can be the same as it was used for chloride, because the moisture movement can carry any other ions as it carries the chloride ions. However, the effect of multispecies on moisture transport cannot be treated in the same way. This is because the effect of the transport of a specific ion on the moisture movement varies with the transport rate of the ion. Therefore, the coupling parameter, δ, of each species can be estimated by the ratio between a specific ionic species and the chloride ion as shown in Equation (17).

(16) $$D_{i-H}=\varepsilon \cdot cl_{f}=\varepsilon \cdot c_{i}$$

(17) $$\frac{D_{i}}{D_{cl}}=\frac{D_{H-i}}{D_{H-cl_{f}}}=\frac{\delta_{i}\cdot C_{i}}{\delta \cdot cl_{f}}$$

## 3. Parallel Finite Element Formulation

### 3.1. Numerical Modeling with Finite Element Method 

In order to solve a coupled hygro-chemo transport problem over time, a large linear system equation with parallel finite element method is derived. The finite element formulation is briefly expressed in this section. The continuous variables in the coupled moisture and chemicals transport equations, free chloride, other species (Na^+^, Ca^2+^, K^+^, OH^−^), and relative humidity ($H_{m}$) are spatially discretized over the space domain, Ω. The domain discretization can be described as shown in Equation (18),

(18) $$\Omega \;=\;\overset{nel}{\underset{e=1}{\cup }}\;\Omega_{e}$$

in which *nel* is the total number of elements in space domain and $\Omega_{e}$ is an element. It is also defined ∂Ω as the boundary of computational domain and $\partial \Omega_{e}$ the boundary of subdomain.

The unknown variables in Equations (19) and (20) are defined in terms of nodal values, $\left\{H_{m}\right\}$ and $\left\{C_{i}\right\}$

(19) $$H_{m}=\left\lfloor N\right\rfloor \left\{{\overset{\wedge }{H}}_{m}\right\}$$

(20) $$C_{i}=\left\lfloor N\right\rfloor \left\{{\overset{\wedge }{C}}_{i}\right\}$$

where, ⌊N⌋ is the triangle element shape function. The notations ⌊⌋ and {} are row and column vectors, respectively. The element shape functions are expressed as following Equation (21),

(21) $$\left\lfloor N\right\rfloor =\left\lfloor N_{1}\;N_{2}\ldots N_{n}\right\rfloor$$

in which *N_i_* is the shape function for node *i* and *n* is the total numbers of nodes in an element. The unknown vectors of relative humidity $\left\{\overset{\wedge }{H_{m}}\right\}$ in Equation (19) and multispecies $\left\{\overset{\wedge }{C_{i}}\right\}$ in Equation (20) can be defined as below,

(22) $$\left\{{\overset{\wedge }{H}}_{m}\}=\{{\overset{\wedge }{H}}_{m1},{\overset{\wedge }{H}}_{m2},{\overset{\wedge }{H}}_{m3},\cdot \cdot \cdot \cdot \cdot \cdot \cdot \cdot \cdot ,{\overset{\wedge }{H}}_{mn}\right\}$$

(23) $$\left\{{\overset{\wedge }{C}}_{i}\}=\{{\overset{\wedge }{C}}_{i1},{\overset{\wedge }{C}}_{i2},{\overset{\wedge }{C}}_{i3},\cdot \cdot \cdot \cdot \cdot \cdot \cdot \cdot \cdot \cdot \cdot ,{\overset{\wedge }{C}}_{in}\right\}$$

The nodal relative humidity, free chloride, and other species concentrations are solved by substituting the approximated values of Equations (19) and (20) into governing equations of Equations (8) and (10), and applying the Galerkin procedure to the weak forms, then the finite element matrix can be obtained as shown in Equation (24):

(24) $$\frac{d}{dt}\left(\left[C_{e}(\overset{\wedge }{\phi })\right]\left\{\overset{\wedge }{\phi }\right\}\right)=\left[K_{e}(\overset{\wedge }{\phi })\right]\left\{\overset{\wedge }{\phi }\right\}$$

where, the element matrices and vector are as following Equations (25)–(27),

(25) $$\left[C_{e}\right]=\left[\begin{array}{cc} C_{h} & 0\\ 0 & C_{i} \end{array}\right]$$

(26) $$\left[K_{e}\right]=\left[\begin{array}{cc} K_{hh} & K_{hi}\\ K_{ih} & K_{ii} \end{array}\right]$$

(27) $$\{\overset{\wedge }{\phi }\}=\left\lfloor {\overset{\wedge }{H}}_{m}\overset{\wedge }{C_{i}}\right\rfloor$$

In detail, the components in element matrices are as Equations (28)–(33),

(28) $$\left[C_{h}\right]=\int_{\Omega_{e}}{\left\lfloor N_{h}\right\rfloor }^{T}C_{h}\left\lfloor N_{h}\right\rfloor d\Omega$$

(29) $$\left[C_{i}\right]=\int_{\Omega_{e}}{\left\lfloor N_{i}\right\rfloor }^{T}C_{i}\left\lfloor N_{i}\right\rfloor d\Omega$$

(30) $$\left[K_{hh}\right]=-\int_{\Omega_{e}}\nabla {\left\lfloor N_{h}\right\rfloor }^{T}D_{Hm}\nabla \left\lfloor N_{h}\right\rfloor d\Omega +\int_{\partial \Omega_{e}}{\left\lfloor N_{h}\right\rfloor }^{T}D_{Hm}\nabla \left\lfloor N_{h}\right\rfloor d\Gamma$$

(31) $$\left[K_{hi}\right]=-\int_{\Omega_{e}}\nabla {\left\lfloor N_{h}\right\rfloor }^{T}D_{H-i}\nabla \left\lfloor N_{i}\right\rfloor d\Omega +\int_{\partial \Omega_{e}}{\left\lfloor N_{h}\right\rfloor }^{T}D_{H-i}\nabla \left\lfloor N_{i}\right\rfloor d\Gamma$$

(32) $$\left[K_{ih}\right]=-\int_{\Omega_{e}}\nabla {\left\lfloor N_{i}\right\rfloor }^{T}D_{i-H}\nabla \left\lfloor N_{h}\right\rfloor d\Omega +\int_{\partial \Omega_{e}}{\left\lfloor N_{i}\right\rfloor }^{T}D_{i-H}\nabla \left\lfloor N_{h}\right\rfloor d\Gamma$$

(33) $$\left[K_{ii}\right]=-\int_{\Omega_{e}}\nabla {\left\lfloor N_{i}\right\rfloor }^{T}D_{i}\nabla \left\lfloor N_{i}\right\rfloor d\Omega +\int_{\partial \Omega_{e}}{\left\lfloor N_{i}\right\rfloor }^{T}D_{i}\nabla \left\lfloor N_{i}\right\rfloor d\Gamma$$

Finally, Equation (34) is also discretized in time space with time interval $\Delta t=t^{{}_{\xi +1}}-t^{{}_{\xi }}$ as following,

(34) $${\left(\left[C_{e}(\overset{\wedge }{\phi })-\theta \cdot \Delta t\cdot K_{e}(\overset{\wedge }{\phi })\right]\left\{\overset{\wedge }{\phi }\right\}\right)}^{\xi +1}={\left(\left[C_{e}(\overset{\wedge }{\phi })-(1-\theta )\cdot \Delta t\cdot K_{e}(\overset{\wedge }{\phi })\right]\left\{\overset{\wedge }{\phi }\right\}\right)}^{\xi }$$

The value of parameter θ is related to the solution method adopted in the program. Typical values of θ are 0, 1/2, and 1 correspond to fully explicit, semi-implicit, and fully implicit methods, respectively. The semi-implicit method called Crank–Nicholson method is used in this study. 

Equation (34) is simplified as linear system equation.

(35) $${\left[A\right]}^{\xi +1}{\left\{\overset{\wedge }{\phi }\right\}}^{\xi +1}={\left\{b\right\}}^{\xi }$$

where,

(36) $${\left[A\right]}^{\xi +1}={\left[C_{e}(\overset{\wedge }{\phi })-\theta \cdot \Delta t\cdot K_{e}(\overset{\wedge }{\phi })\right]}^{\xi +1}$$

(37) $${\left\{b\right\}}^{\xi }={\left(\left[C_{e}(\overset{\wedge }{\phi })-(1-\theta )\cdot \Delta t\cdot K_{e}(\overset{\wedge }{\phi })\right]\left\{\overset{\wedge }{\phi }\right\}\right)}^{\xi }$$

### 3.2. Overlapping Domain Decomposition Method

The parallel program developed in this study is designed to be divided into multiple fragments that can be executed simultaneously on its own processor with applying a domain decomposition method.

The domain decomposition (DD) method is attractive in parallel finite element computational algorithm because it is usually used for solving large-scale system equations and allows individual subdomain operations to be performed concurrently on separate processors [21].

There are two types of domain decomposition methods, overlapping and nonoverlapping methods. In this study, the overlapping method was applied to solve the linear sparse matrix because of easier implementation in algebraic approach as well as faster convergence than nonoverlapping DD method. Furthermore, the boundaries of extended subdomains are smoother than nonoverlapping subdomains. The domain decomposition method needs to use iterative solver. In this study for the iterative solver, GMRES (Generalized Minimal Residual method) was mainly adopted because of its ability to solve nonsymmetric linear system of global and local matrix. To improve the convergence of multispecies transport problem, the additive Schwarz preconditioner was applied as well.

The details of preconditioned Schwarz method as the mathematical formulations of the overlapping domain decomposition (DD) method can be demonstrated as in the following [34,35]. To obtain an overlapping decomposition of the domain, each subdomain $\Omega_{i}$ can be extend to a larger domain $\Omega_{i}^{\prime }$, that is, $\Omega_{i}\subset \Omega_{i}^{\prime }$. The overlap is uniform and $V_{i}\subset V^{h}$ is the unusual finite element space over $\Omega_{i}^{\prime }$. So, Equation (18) can be rewritten as Equation (38),

(38) $$\Omega \;=\;\overset{nel}{\underset{e=1}{\cup }}\;\Omega_{e}^{\prime }$$

and

(39) $$V^{h}=V_{1}+\cdots +V_{N}$$

Here, $V^{h}$ is a finite dimensional subspace of the Sobolev space V, subscript N is number of subspace, and superscript h is element of size.

Recall Equation (35) and it can be simplified as below,

(40) Au=f

which is usually not well-conditioned. Thus, a good preconditioner should be needed for the success of any iterative method used to solve it.

The additive Schwarz preconditioned system can be written as

(41) $$M^{-1}Au\equiv (T_{1}+\cdots +T_{N})u=g$$

$T_{i}$ is the operators and can be expressed in matrix form as $T_{i}=M^{-1}A=R_{i}^{T}A_{i}^{-1}R_{i}A$, where $R_{i}$ is the restriction matrix and $A_{i}^{-1}$ is the subdomain problem solver. In practice, for the additive algorithm, usually let the number of subdomains, N, be equal to the number of available processors.

In order to form the algorithmic structure of overlapping DD method, there are four components: subgrid preparation component, subdomain solver component, data exchange component, and global administration component. In detail, the starting point of the overlapping DD method is a set of overlapping subdomains, which can be its subdomain solvers. Then for updating the boundary condition during each iteration, data must be exchanged between neighboring subdomains. In order to coordinate the subdomain solvers and data exchange, a global administration component would play a role in controlling the progress of DD iterations, monitoring the global convergence behavior, starting the subdomain solvers, and invoking data exchange [35].

### 3.3. Implementation of Parallel Finite Element Method

For the implementation of a parallel finite element (PFE) program for the multispecies transport problem, various programs were used such as Triangle for mesh generation, Parmetis, PETSc, and MPI [36,37,38,39,40,41]. Parallel finite element program is significantly different from the traditional paradigm for a serial program [22,23,42,43,44,45]. For a parallel computing environment, one of important concerning is to maintain the performing balance of all participating processors and to minimize communication time among the processors. In this study, the developed PFE program for multispecies transport problem employs the single-program-multiple-data (SPMD) paradigm as well as domain decomposition techniques. Each processor of the parallel machine solves a partitioned subdomain, and data communications among subdomains are performed through message passing. The mesh of triangle elements is generated by Triangle which is a program to create exact delaunay triangulations, and it is suitable for finite element analysis [46].

In order to provide the numerical codes involving the implicit numerical solution of PDEs, PETSc (3.0.0 p8) was employed. PETSc library is written in C, but it may be accessed from user codes written in C, Fortran, and C++ [47]. MPI (Message Passing Interface) is a standardized and portable message-passing system designed to function on a wide variety of parallel computers. For reducing the time spent in inter-process communication by computing mesh decompositions, ParMETIS includes routines based on a parallel graph-partitioning algorithm that is especially suited for parallel computations and large-scale numerical simulations involving unstructured meshes [40]. 

**Figure 1:**
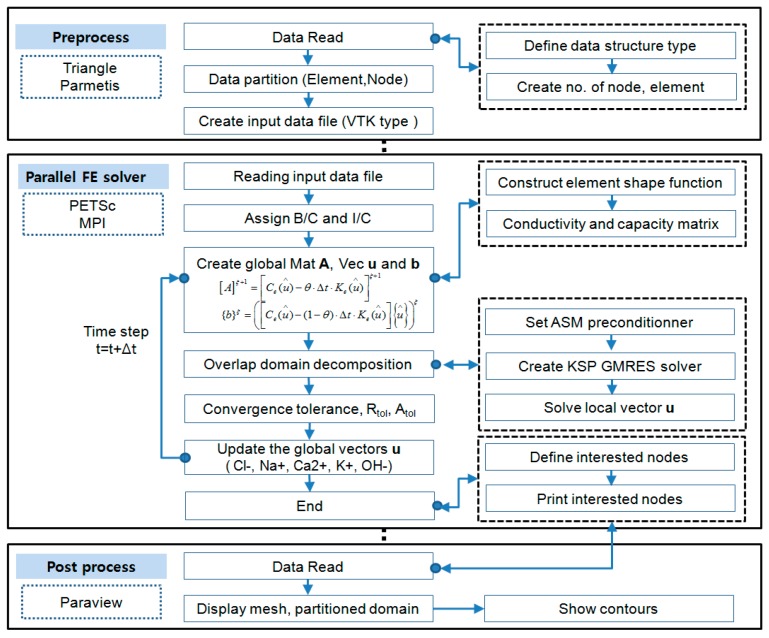
Framework of parallel finite element method based on PETSc (Portable, Extensible Toolkit for Scientific Computation) and Massage Passing Interface (MPI).

In order to capture the transport phenomenon of multi-ions in concrete, a large number of meshes are needed within a thin layer from the top surface to steel rebar of concrete structures. The information of nodes and elements are created by Triangle specialized for creating two-dimensional finite element meshes. To visualize and check the mesh sizes and shapes, ParaView (ver.5.5.2) was employed. This is an open-source multiplatform application for scientific visualization developed to analyze extremely large datasets using distributed memory computing resources. For large-scale information, VTK file format was used because it is easy to be read and written by hand or programmatically. VTK input file is automatically generated once the program runs. Figure 1 shows the schematic configuration of the parallel computational program including pre- and post-processes and parallel FE solver.

## 4. Numerical Results 

### 4.1. Validation of Parallel Finite Element Model 

#### 4.1.1. Test Plan for Deicing Solution Ponding Test 

**Figure 2:**
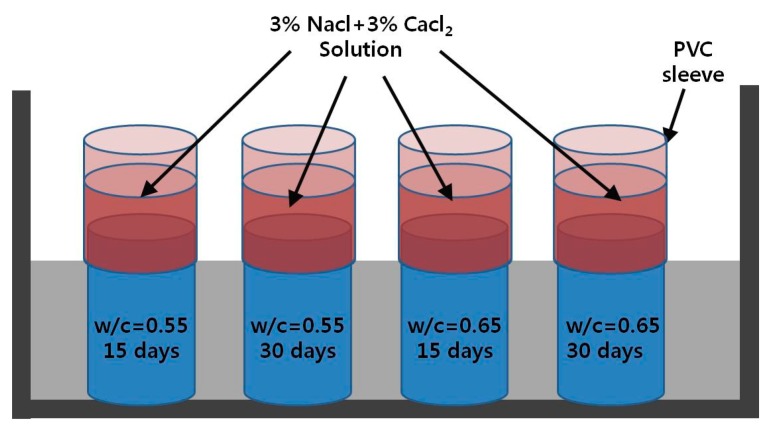
Experimental plan for penetration of deicing salt.

For verifying the parallel finite element algorithm, the test results of multispecies penetration into saturated concrete were employed, which were obtained by Damrongwiriyanupap [48]. The mix proportions used in the experiment were two different water–cement ratios, 0.55 and 0.65. All specimens were immersed in water for 30 days to reach the saturated condition. This is to exclude the effect of moisture gradient. In order to substantialize the multispecies ingress into concrete, the ponding reservoir was designed on the top surface of concrete specimen with plastic sleeve and was filled with multi-types deicing salt composed of 3% of sodium chloride and 3% of calcium chloride solution. To prevent the leakage of deicing solution, steel band clamp and silicone gel were used to tighten the plastic sleeve and concrete specimen as shown in Figure 2. 

#### 4.1.2. Comparison of Numerical Results with Test Results 

The Nernst–Planck equation can be used to characterize the penetration of multi-types of deicing solutions into concrete. In order to verify the numerical model, total chloride profiles corresponding to Table 1 were compared with numerical results [48]. 

**Figure 3:**
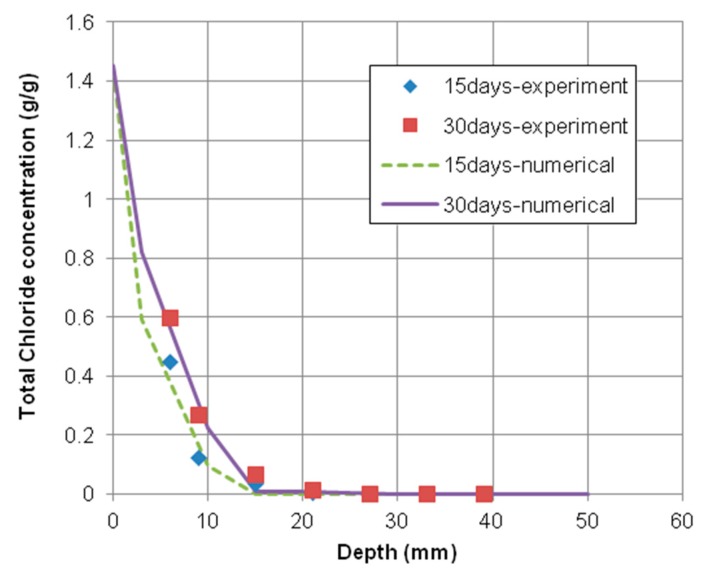
Chloride profiles of concrete specimen with 0.55 water–cement ratio. Exposed for 15 days and 30 days.

**Figure 4:**
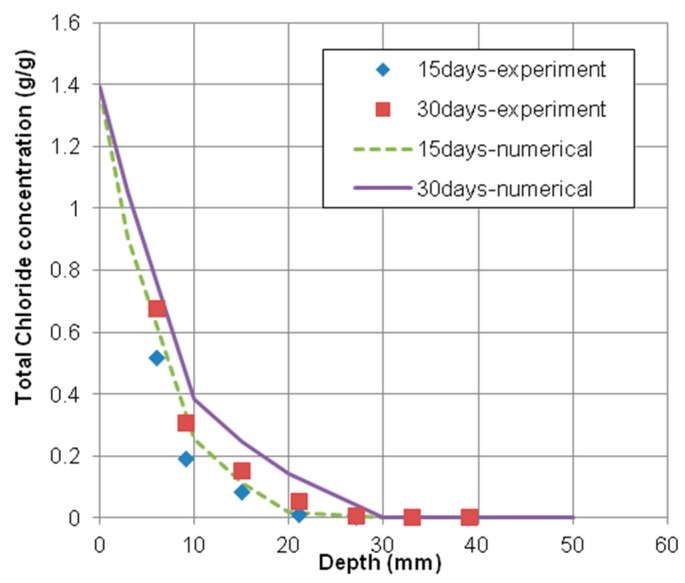
Chloride profiles of concrete specimen with 0.65 water–cement ratio. Exposed for 15 days and 30 days.

As shown in Figure 3 and Figure 4, the time-dependent chloride concentration of concrete specimens casted with 0.55 and 0.65 water–cement ratios was plotted in comparison with experimental and numerical results exposed for 15 and 30 days, respectively. 

These figures indicated that the numerical results have a good agreement with the experimental results of total chloride concentrations. However, the numerical results with 0.55 water–cement ratio fit better with the corresponding experimental results than the case of 0.65 water–cement ratio. That is because the material models used in the numerical analysis were more accurate to predict the penetration of chloride into concrete made by 0.55 water–cement ratio.

**Figure 5:**
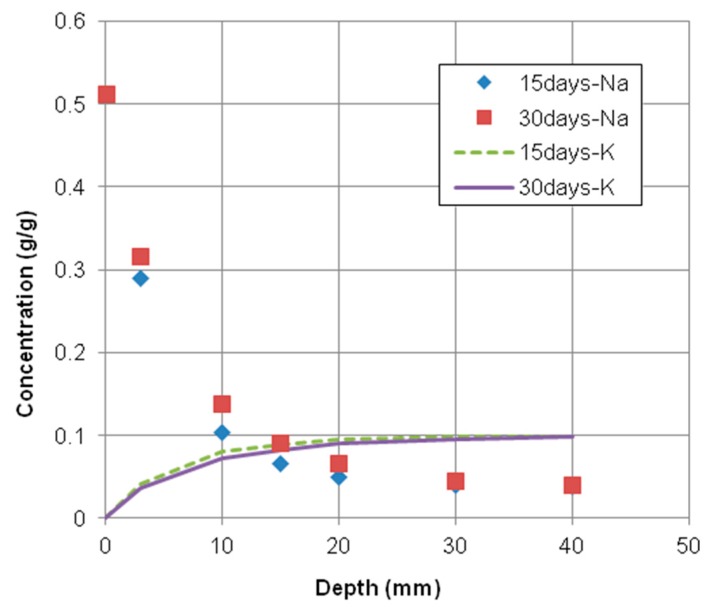
Cation concentration profiles of concrete specimen with 0.55 water–cement ratio. Exposed for 15 days and 30 days.

**Figure 6:**
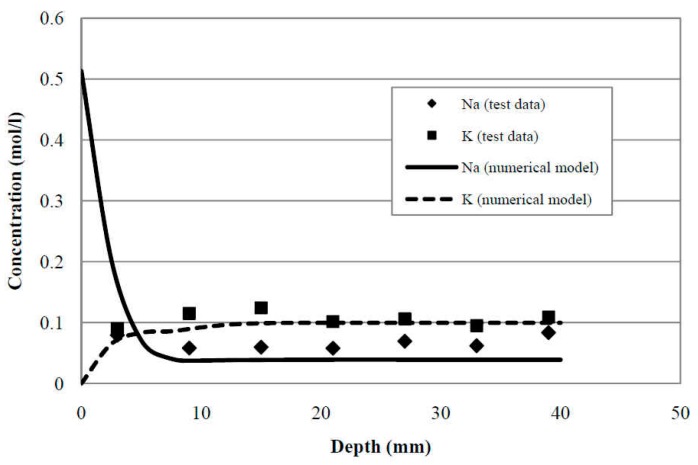
Comparison of test and numerical results. The specimen was exposed for 15 days and 30 days [48].

The present numerical model can be used to simulate not only the chloride transport but also the transport of other chemical species. As illustrated in Figure 5, the concentration profiles of measured sodium (Na^+^) and potassium (K^+^) were plotted in comparison with numerical results at 15 and 30 days of exposure periods. The trends of sodium (Na^+^) and potassium (K^+^) profiles resulting from the prediction model have a good match with the experimental results as shown in Figure 6. Therefore, this numerical model with the parallel finite element algorithm is validated by the experimental results and can be used to predict the multispecies ingress into concrete under saturated condition.

### 4.2. Prediction for Multispecies Transport in Nonsaturated Concrete

#### 4.2.1. Numerical Example

**Figure 7:**
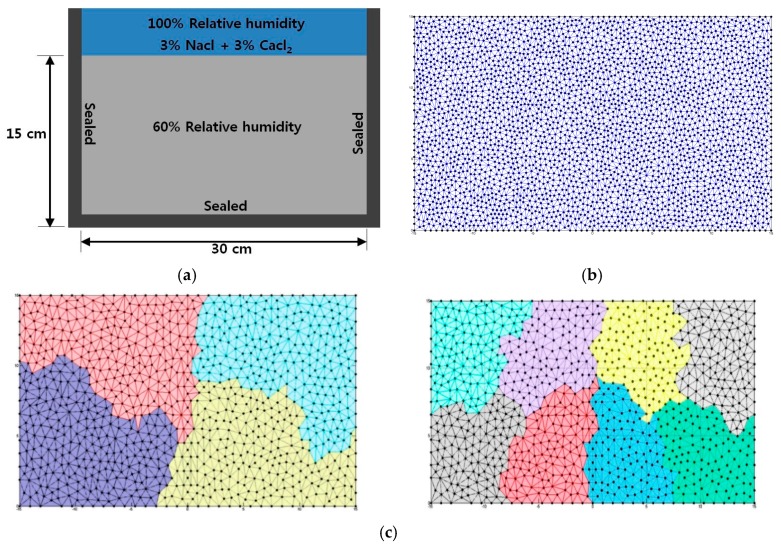
Numerical simulation model and partitioning meshes. (**a**) Geometry information; (**b**) Mesh generation; (**c**) Partitioning meshes with 4 and 8 processors.

This study focused on the chloride penetrating into concrete specimen from the top surface. The dimension of the specimen was 15 cm high and 30 cm wide as shown in Figure 7a. The top surface of concrete specimen was exposed to the solution of 3% of NaCl and 3% of CaCl_2_, and the other species, Na^+^, K^+^, and OH^−^, were initialized to be dissolved in the concrete pore solution. Particularly, the concentration of alkali ions in pore solution, Na+ and K^+^, was obtained from cement manufacturer which provided the chemical composition of cement [48]. In reality, not only single type of deicing salt is used but also different combinations of deicing salts are frequently used at the same location. Therefore, in this example, the combination of 3% of NaCl and 3% of CaCl_2_ was used as the boundary condition. All other sides except the top surface were assumed to be sealed. The moisture condition inside the concrete specimen was assumed to be partially saturated with 60% relative humidity (RH). 

Obviously, this numerical model is used to simulate the concrete deterioration with multispecies under service condition in which the concrete is not saturated, regarding to a long-term pond test. With these initial and boundary conditions, one can speculate that the chloride and the moisture transports were in the same direction. Total analysis time was 400 days and the time step was 0.5 day. There were two kinds of stopping tolerance conditions used in the program; relative tolerance, R_tol_ = 10^−4^, and absolute tolerance, A_tol_ = 10^−10^, respectively. As previously mentioned, Krylov Subspace Method (KSP) GMRES with additive Schwarz preconditioner was used to solve the large linear sparse matrix.

Figure 7b shows the meshes of numerical domain which approximately consist of 3000 nodes and 6000 triangles. The whole mesh was partitioned into 4 and 8 sub-meshes as shown in Figure 7c. Moreover, each sub-mesh had almost the same number of elements. Furthermore, the number of nodes on subdomain mesh boundaries was at its minimal.

In order to implement the parallel finite element computation for coupled moisture and multispecies transport problem, large sparse matrix should be derived and the continuous variables, relative humidity ($H_{m}$), and other 5 species (Na, Ca, OH, K, and Cl), in Nernst–Plank equations, were spatially discretized over the space domain, Ω. In order to solve the numerical model in time space, the semi-implicit method called Crank–Nicholson method was used in this study.

The material parameters and input data for the numerical simulations were shown in Table 1. These were diffusion coefficients, initial concentrations at the top surface, and initial concentrations in concrete pore solution of each ionic species, water-to-cement ratio, and volume fraction of aggregate. The numerical simulations were performed in nonsaturated condition. This means that the relative humidity inside and outside of the concrete are different. The units of all ionic concentrations were in mol/liter except for the total chloride which is in grams of chloride/gram of concrete weight (g/g).

#### 4.2.2. Performance of Parallel Finite Element Model

To verify the performance of parallel finite element algorithm, the concept of speed-up and efficiency is necessary. The definition of speed-up is the ratio between the time taken by the code to execute on a single processor, *Ts*(*n*), and the time taken for the same code to execute on p processors, *Tp*(*n*), as below Equation (42). Parallel efficiency can be defined as the ratio between speed-up and the number of processors as shown in Equation (43).

(42) $$S_{P}(n)=\frac{T_{S}(n)}{T_{P}(n)}$$

(43) $$E_{P}(n)=\frac{S_{P}(n)}{P}$$

**Figure 8:**
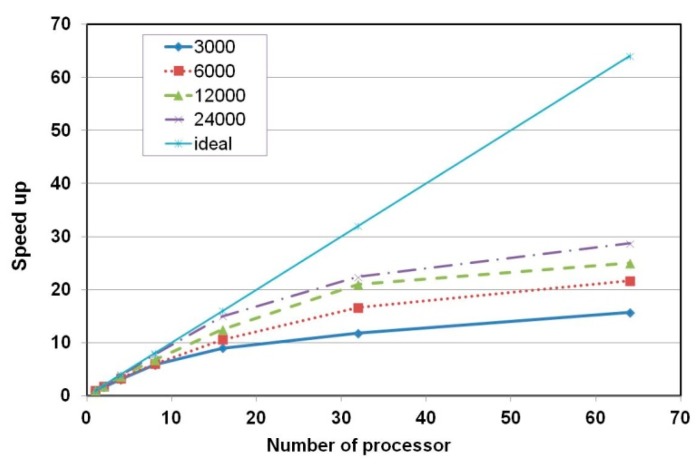
Speed-up according to the number of nodes and processors.

**Figure 9:**
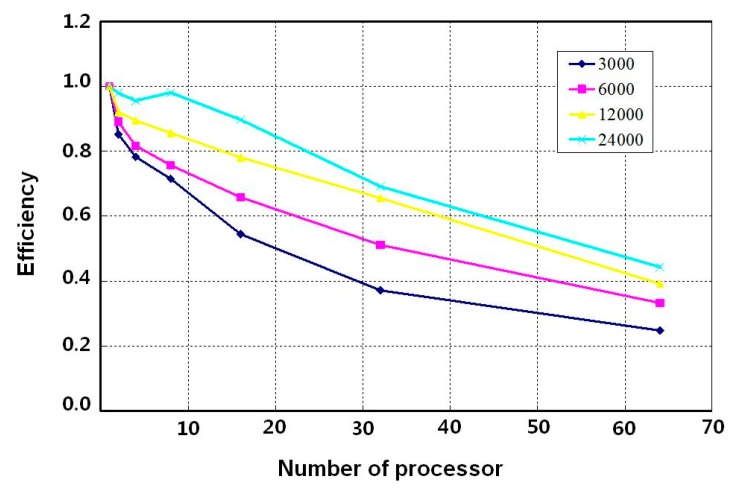
Efficiency according to the number of nodes and processors.

For the parallel effect of this algorithm, the meshes composed of the numbers of nodes, 3000, 6000, 12,000, and 24,000, were used with increasing the number of processors up to 64. In Figure 8, as the number of processors increases, the analysis size of problem assigned on each processor decreases and the speed-up gradually increases. For example, in case of 24,000 nodes, the speed-up with 8 processors is close to ideal speed-up and the efficiency is almost 1 as shown in Figure 9. On the other hand, in case of 3000 nodes, the speed-up tends to be slowly increased as solving the problem with more than 2 processors because of the communication time consumption between processors.

#### 4.2.3. Numerical Results of Multispecies Transport in Nonsaturated Concrete

In the nonsaturated concrete, the effect of chloride penetration on moisture transport is significant [32]. For observing the penetration of chloride into concrete from the top surface, the deicing salt combined with NaCl and CaCl_2_ was used as a boundary condition. Moreover, the ions in concrete pore solution, K^+^, Na^+^, and OH^−^, were considered as initial conditions. The material parameters and input data for numerical simulations were the same as used in Table 2. The numerical simulations were performed in nonsaturated condition meaning the moisture gradient (RH = 60% inside and RH = 100% outside of concrete) for its effect on the diffusion of ionic species in concrete. Thus, the moisture gradient was from outside to inside concrete. That is, the concrete specimen was exposed to 3% NaCl and 3% CaCl_2_ solutions on the top surface.

**Figure 10:**
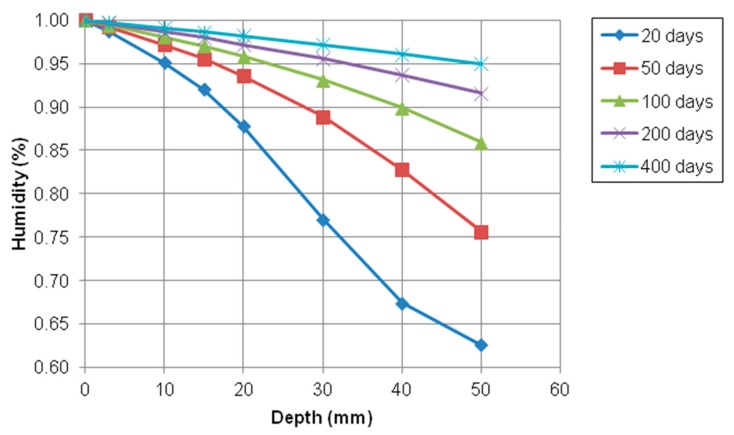
Profile of relative humidity at different depth over time.

Figure 10 shows the humidity profile at 20, 50, 100, 200, and 400 days under aggressive chemical condition. It could be observed that the moisture gradient moved from the exposed surface to inside concrete and it finally converged to 100% and remained the steady-state status unchanging in time. In fact, the moisture gradient could help to transport chloride, sodium, and calcium ions and simultaneously accelerate the penetration rate of these ions.

**Figure 11:**
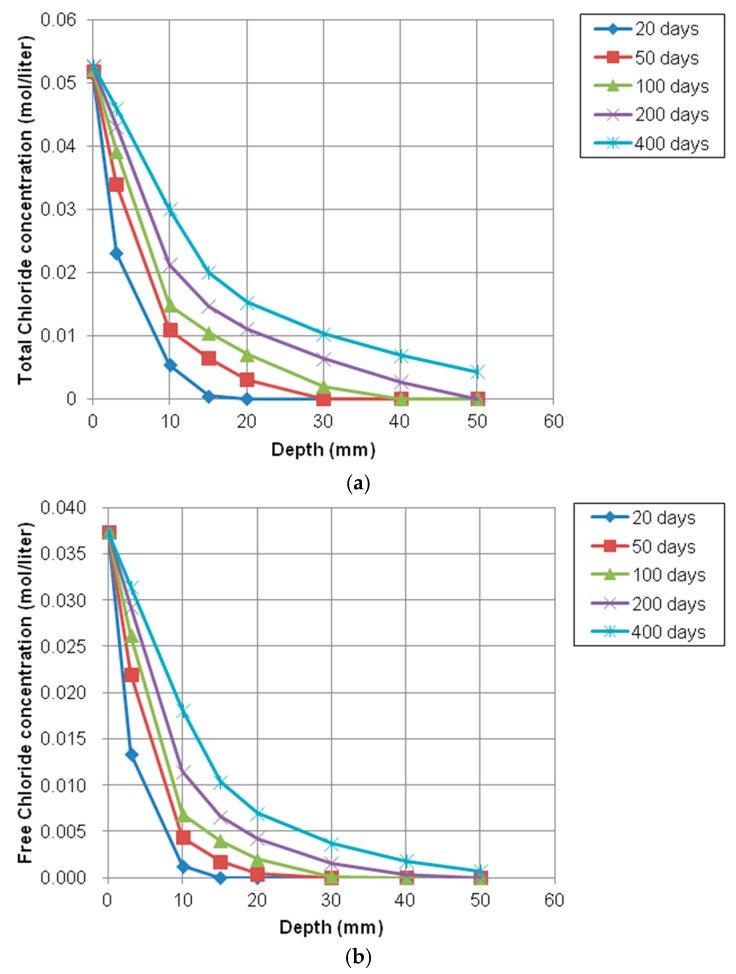
Profile of chloride concentration at different depth over time. (**a**) Total chloride concentration profile over time; (**b**) Free chloride concentration profile over time.

The profile of total and free chloride concentration at different exposure times is shown in Figure 11. The depth of penetration was measured from the top surface of concrete specimen. As seen in Figure 11, the total and free chloride concentrations were decreased with increasing the depth from the top surface. As the exposure time goes on, at the same depth, the chloride concentration is getting higher because free and bound chlorides tend to increase with longer times of exposure. 

**Figure 12:**
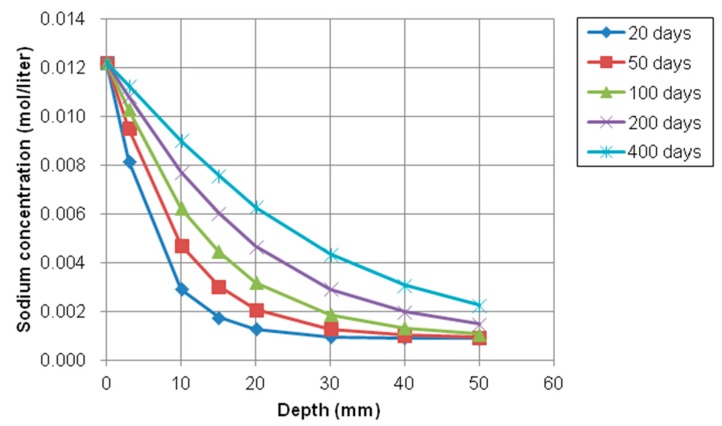
Profile of sodium concentration at different depth over time.

**Figure 13:**
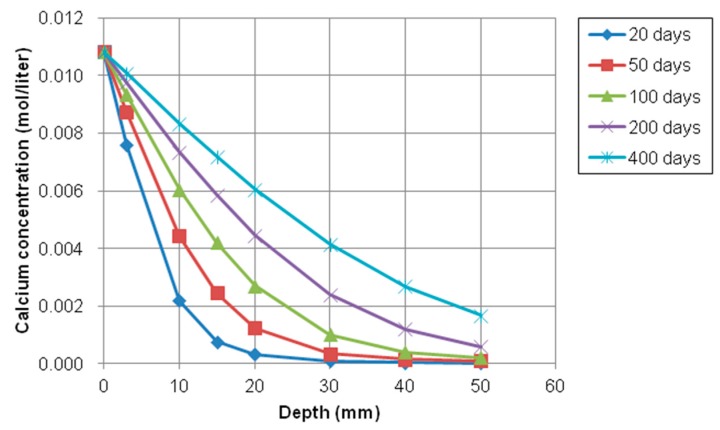
Profile of calcium concentration at different depth over time.

As plotted in Figure 12 and Figure 13, the trends of sodium and calcium concentration were similar to chloride concentration because the concentration of the exposed surface was higher than inner concentration and the concentration gradient could accelerate the movement of ions (Cl^−^, Na^+^, and Ca^2+^). That is, the ionic transport can occur through saturated and partially saturated concrete. Moisture transport in saturated condition can be controlled by a pressure gradient into concrete. The ionic species dissolved in pore solution also can be moved by permeation of moisture or water.

**Figure 14:**
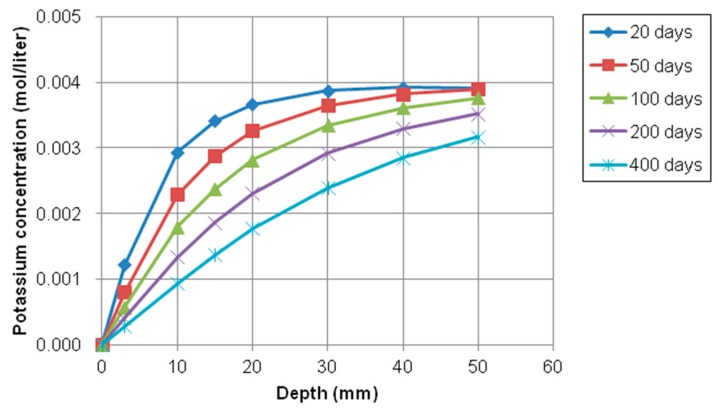
Profile of potassium concentration at different depth over time.

**Figure 15:**
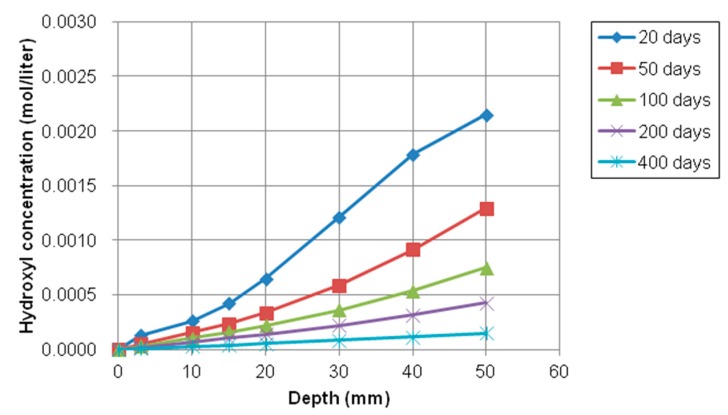
Profile of hydroxyl concentration at different depth over time.

On the contrary of chloride, sodium, and calcium concentration profiles, the concentration gradient of potassium (K^+^) and hydroxyl (OH^−^) ion were from inside to outside of concrete because the inside concentration was higher than the concentration of the exposed surface, which means that any source of potassium and hydroxyl solution was not supplied on the top surface. The variations of potassium and hydroxyl concentration with depth at different exposure periods were shown in Figure 14 and Figure 15, respectively. 

As shown in Figure 14 and Figure 15, the concentration of potassium and hydroxyl ions increases with the increase of depth from the top surface. However, these values decrease with increasing of exposure times. The gradient of relative humidity inside and outside concrete is initially high. However, as exposure time goes by, the gradient and slope of between internal and external decrease and nonlinearity is reduced. For instance, the difference in concentration of potassium and hydroxyl at between 20 days and 50 days is much higher and clearer than difference of concentration at between 200 and 400. It is noticed that moisture has influence on the transport of multi-ionic species in concrete by accelerating the penetration rate of multispecies. 

## 5. Conclusions

The chloride-induced corrosion of steel reinforcement is undoubtedly one of the most important causes and has an effect on the integrity and the service life of concrete structures. The chloride as well as other ionic species (Na^+^, Ca^2+^, K^+^, OH^−^) come from deicing salt and are transported from the environment into concrete. The phenomenon of multi-ions transport can be observed in a thin layer (concrete cover depth) of concrete structures. Parallel computing is one of effective techniques to save computational time. To investigate the transport mechanism of multispecies in this study, complex scientific methods and more accurate mathematical models are needed.

1. The parallel finite element model was implemented with higher level library, PETSc (Portable, Extensible Toolkit for Scientific Computation) and MPI (Massage Passing Interface), based on C++ program language. For the easier implementation in algebraic approach as well as faster convergence, the overlapping DD method was employed to solve the linear sparse matrix. To improve the convergence of multispecies transport problem, the additive Schwarz preconditioner was applied and GMRES (Generalized Minimal Residual method) as an iterative solver was mainly adopted because of its ability to solve nonsymmetric linear system of global and local matrix.
2. The new mathematical model was developed based on the Nernst–Planck equation and null current condition to solve the ionic-induced electrostatic potential. This model included the material models of transport mechanisms in cement paste and aggregates. The model can be used to simulate the multispecies penetration into concrete structures by considering the effect of moisture gradient.
3. Moisture transport has significant effect on penetration of deicing salts into concrete by accelerating the penetration rate of chloride, sodium, and calcium ions. The coupling parameter was assumed because of no experimental data or material models of coupling parameters for other species. The parameter considering the effect of moisture on ionic transport was assumed by the same as used for the chloride ions due to carrying any other ions as it carries the chloride ions. The coupling parameter of each species can be estimated by the ratio between a specific ionic species and the chloride ions.
4. In order to verify the new mathematical model, the parallel finite element program was developed and the numerical results were compared with the experimental results about the penetration of multi-types of deicing solutions into saturated concrete. The comparisons showed good results and the accuracy of the new mathematical model for the transport mechanism of the multispecies was proven effectively.
5. The model prediction for the ionic transport in partially saturated concrete was conducted to investigate the transport of ionic species such as chloride, sodium, calcium, hydroxyl, and potassium. The penetration of chloride, sodium, and calcium increased over time because the initial concentration outside concrete was much higher and the moisture gradient had an effect on accelerating the transport rate of ions. However, the concentration of hydroxyl and potassium was decreased due to opposite reason. This model can be used satisfactorily to predict the penetration of aggressive chemicals, such as deicing salts into nonsaturated condition.
6. For more robust verification, additional deicing ponding tests under nonsaturated conditions are needed in the future. More material models are needed to take into account the effects of mineral additives such as fly ash and silica fume on chloride penetration and ion transport in concrete. Additionally, the temperature effect is to be considered as one of the crucial parameters.

## Figures and Tables

**Table 1:** Total chloride profile associated with two different water–cement ratio and time period on depth.

W/C	Time (days)	Depth (mm)						
		0–6	6–12	12–18	18–24	24–30	30–36	36–42
0.55	15	0.450	0.125	0.036	0.006	0.003	0.003	0.002
	30	0.599	0.269	0.068	0.014	0.004	0.003	0.002
0.65	15	0.517	0.193	0.085	0.011	0.004	0.002	0.002
	30	0.677	0.31	0.153	0.055	0.008	0.003	0.003

**Table 2:** Material parameters and input data for numerical analysis.

Species		Unit	K	Na	Cl	OH	Ca
Charge number			+1	+1	−1	−1	+2
Diffusion coefficient		m^2^/s	4.0 × 10^−11^	2.8 × 10^−11^	Refer to Equation (14)	5.3 × 10^−10^	1.6 × 10^−11^
Initial condition	at top surface	mol/L	0	0.513	1.053	0	0.27
	in pore solution	mol/L	0.0995	0.0389	0	0.1384	0
Cement type			I/II				
Water–cement ratio			0.55				
Volume fraction of aggregate			0.65				
Outside RH		%	100				
Inside RH		%	60				

## Appendix A

### Derivation of Diffusivity Matrix

The physical problems such as heat transfer, fluid flow, and mass transport can be explained in terms of partial differential equations (PDE). These PDE can be approximately solved by numerical methods such as finite element method or finite difference method. Finite element method is an effective tool to solve the governing equation for the transport problem and it is widely used to predict the chloride transport and the moisture movement in concrete. The transport phenomena of multispecies in concrete are described with the Nernst–Plank equation. Not only transport mechanism due to concentration gradient but also the electrical coupling between the different ions due to the deicing salt and pore solution in the concrete is accounted for the Nernst–Planck equation.

In order to develop the finite element program, first of all, the space domain, Ω, is discretized into a large number of finite elements as mentioned in main text body. If a problem is time-dependent, it is considered as ordinary differential equation and time need to be discretized.

The fluxes of multispecies in the pore solution of saturated concrete are derived from Equation (1) and the transport mechanism for a given time period is expressed with Equation (7) as called the mass balance equation.

In order to solve the system of equations, a supplemental relation is needed to consider the electrical potential due to inherently caused by the movement of all species. In this study, null current condition is used as expressed in Equation (3).

From the null current condition, Equation (1) is put into Equation (3) and we have

(A1) $$\sum_{i=1}^{n}z_{i}\left(-D_{i}\nabla C_{i}-z_{i}D_{i}\left(\frac{F}{RT}\nabla \phi \right)C_{i}\right)=0$$

And then,

(A2) $$\frac{F}{RT}\nabla \phi =-\frac{\sum_{i=1}^{n}z_{i}D_{i}\nabla C_{i}}{\sum_{i=1}^{n}z_{i}^{2}D_{i}C_{i}}$$

The governing equation is

(A3) $$\frac{\partial C_{i}}{\partial t}\;=\;\nabla \left(D_{i}\nabla C_{i}+z_{i}D_{i}\left(\frac{F}{RT}\nabla \phi \right)C_{i}\right)=\nabla \left(D_{i}\nabla C_{i}+z_{i}D_{i}\left(-\frac{\sum_{j=1}^{n}z_{j}D_{j}\nabla C_{j}}{\sum_{j=1}^{n}z_{j}^{2}D_{j}C_{j}}\right)C_{i}\right)$$

Let us consider three different ions in concrete,

In the case of *i* = 1,

(A4) $$\begin{array}{ll} \frac{\partial C_{1}}{\partial t} & =\frac{\partial }{\partial x}\left\{D_{1}\cdot \frac{\partial C_{1}}{\partial x}+z_{1}\cdot D_{1}\cdot C_{1}\cdot \left(-\frac{\sum_{i=1}^{3}z_{i}\cdot D_{i}\cdot \frac{\partial C_{i}}{\partial x}}{\sum_{i=1}^{3}z_{i}^{2}\cdot D_{i}\cdot C_{i}}\right)\right\}\\ & =\frac{\partial }{\partial x}\left\{D_{1}\cdot \frac{\partial C_{1}}{\partial x}-z_{1}\cdot D_{1}\cdot C_{1}\cdot \left(\frac{z_{1}\cdot D_{1}\cdot \frac{\partial C_{1}}{\partial x}+z_{2}\cdot D_{2}\cdot \frac{\partial C_{2}}{\partial x}+z_{3}\cdot D_{3}\cdot \frac{\partial C_{3}}{\partial x}}{\sum_{i=1}^{3}z_{i}^{2}\cdot D_{i}\cdot C_{i}}\right)\right\}\\ & =\frac{\partial }{\partial x}\left\{\begin{array}{l} D_{1}\cdot \frac{\partial C_{1}}{\partial x}-z_{1}\cdot D_{1}\cdot C_{1}\cdot \frac{z_{1}\cdot D_{1}}{\sum_{i=1}^{3}z_{i}^{2}\cdot D_{i}\cdot C_{i}}\cdot \frac{\partial C_{1}}{\partial x}\\ -z_{1}\cdot D_{1}\cdot C_{1}\cdot \frac{z_{2}\cdot D_{2}}{\sum_{i=1}^{3}z_{i}^{2}\cdot D_{i}\cdot C_{i}}\cdot \frac{\partial C_{2}}{\partial x}-z_{1}\cdot D_{1}\cdot C_{1}\cdot \frac{z_{3}\cdot D_{3}}{\sum_{i=1}^{3}z_{i}^{2}\cdot D_{i}\cdot C_{i}}\cdot \frac{\partial C_{3}}{\partial x} \end{array}\right\} \end{array}$$

In the case of *i* = 2,

(A5) $$\begin{array}{ll} \frac{\partial C_{2}}{\partial t} & =\frac{\partial }{\partial x}\left\{D_{2}\cdot \frac{\partial C_{2}}{\partial x}+z_{2}\cdot D_{2}\cdot C_{2}\cdot \left(-\frac{\sum_{i=1}^{3}z_{i}\cdot D_{i}\cdot \frac{\partial C_{i}}{\partial x}}{\sum_{i=1}^{3}z_{i}^{2}\cdot D_{i}\cdot C_{i}}\right)\right\}\\ & =\frac{\partial }{\partial x}\left\{\begin{array}{l} D_{2}\cdot \frac{\partial C_{2}}{\partial x}-z_{2}\cdot D_{2}\cdot C_{2}\cdot \frac{z_{1}\cdot D_{1}}{\sum_{i=1}^{3}z_{i}^{2}\cdot D_{i}\cdot C_{i}}\cdot \frac{\partial C_{1}}{\partial x}\\ -z_{2}\cdot D_{2}\cdot C_{2}\cdot \frac{z_{2}\cdot D_{2}}{\sum_{i=1}^{3}z_{i}^{2}\cdot D_{i}\cdot C_{i}}\cdot \frac{\partial C_{2}}{\partial x}-z_{2}\cdot D_{2}\cdot C_{2}\cdot \frac{z_{3}\cdot D_{3}}{\sum_{i=1}^{3}z_{i}^{2}\cdot D_{i}\cdot C_{i}}\cdot \frac{\partial C_{3}}{\partial x} \end{array}\right\} \end{array}$$

In the case of *i* = 3,

(A6) $$\begin{array}{ll} \frac{\partial C_{3}}{\partial t} & =\frac{\partial }{\partial x}\left\{D_{3}\cdot \frac{\partial C_{3}}{\partial x}+z_{3}\cdot D_{3}\cdot C_{3}\cdot \left(-\frac{\sum_{i=1}^{3}z_{i}\cdot D_{i}\cdot \frac{\partial C_{i}}{\partial x}}{\sum_{i=1}^{3}z_{i}^{2}\cdot D_{i}\cdot C_{i}}\right)\right\}\\ & =\frac{\partial }{\partial x}\left\{\begin{array}{l} D_{3}\cdot \frac{\partial C_{3}}{\partial x}-z_{3}\cdot D_{3}\cdot C_{3}\cdot \frac{z_{1}\cdot D_{1}}{\sum_{i=1}^{3}z_{i}^{2}\cdot D_{i}\cdot C_{i}}\cdot \frac{\partial C_{1}}{\partial x}\\ -z_{3}\cdot D_{3}\cdot C_{3}\cdot \frac{z_{2}\cdot D_{2}}{\sum_{i=1}^{3}z_{i}^{2}\cdot D_{i}\cdot C_{i}}\cdot \frac{\partial C_{2}}{\partial x}-z_{3}\cdot D_{3}\cdot C_{3}\cdot \frac{z_{3}\cdot D_{3}}{\sum_{i=1}^{3}z_{i}^{2}\cdot D_{i}\cdot C_{i}}\cdot \frac{\partial C_{3}}{\partial x} \end{array}\right\} \end{array}$$

We can rearrange Equation (A4)–(A6) in matrix form,

(A7) $$\left\{\begin{array}{c} \frac{\partial C_{1}}{\partial t}\\ \frac{\partial C_{2}}{\partial t}\\ \frac{\partial C_{3}}{\partial t} \end{array}\right\}=\frac{\partial }{\partial x}\cdot \left[\begin{array}{ccc} D_{1}-z_{1}\cdot D_{1}\cdot C_{1}\cdot \frac{z_{1}\cdot D_{1}}{\sum_{i=1}^{3}z_{i}^{2}\cdot D_{i}\cdot C_{i}} & -z_{1}\cdot D_{1}\cdot C_{1}\cdot \frac{z_{2}\cdot D_{2}}{\sum_{i=1}^{3}z_{i}^{2}\cdot D_{i}\cdot C_{i}} & -z_{1}\cdot D_{1}\cdot C_{1}\cdot \frac{z_{3}\cdot D_{3}}{\sum_{i=1}^{3}z_{i}^{2}\cdot D_{i}\cdot C_{i}}\\ -z_{2}\cdot D_{2}\cdot C_{2}\cdot \frac{z_{1}\cdot D_{1}}{\sum_{i=1}^{3}z_{i}^{2}\cdot D_{i}\cdot C_{i}} & D_{2}-z_{2}\cdot D_{2}\cdot C_{2}\cdot \frac{z_{2}\cdot D_{2}}{\sum_{i=1}^{3}z_{i}^{2}\cdot D_{i}\cdot C_{i}} & -z_{2}\cdot D_{2}\cdot C_{2}\cdot \frac{z_{3}\cdot D_{3}}{\sum_{i=1}^{3}z_{i}^{2}\cdot D_{i}\cdot C_{i}}\\ -z_{3}\cdot D_{3}\cdot C_{3}\cdot \frac{z_{1}\cdot D_{1}}{\sum_{i=1}^{3}z_{i}^{2}\cdot D_{i}\cdot C_{i}} & -z_{3}\cdot D_{3}\cdot C_{3}\cdot \frac{z_{2}\cdot D_{2}}{\sum_{i=1}^{3}z_{i}^{2}\cdot D_{i}\cdot C_{i}} & D_{3}-z_{3}\cdot D_{3}\cdot C_{3}\cdot \frac{z_{3}\cdot D_{3}}{\sum_{i=1}^{3}z_{i}^{2}\cdot D_{i}\cdot C_{i}} \end{array}\right]\cdot \left\{\begin{array}{c} \frac{\partial C_{1}}{\partial x}\\ \frac{\partial C_{2}}{\partial x}\\ \frac{\partial C_{3}}{\partial x} \end{array}\right\}$$

As applying Galerkin’s method to the residual functions in Equation (A7) and discretizing it in time space, the chloride concentration can be obtained.
